# An overview of methodological approaches in systematic reviews

**DOI:** 10.1111/jebm.12468

**Published:** 2022-04-13

**Authors:** Prabhakar Veginadu, Hanny Calache, Mark Gussy, Akshaya Pandian, Mohd Masood

**Affiliations:** ^1^ Department of Rural Clinical Sciences, La Trobe Rural Health School La Trobe University Bendigo Victoria Australia; ^2^ Lincoln International Institute for Rural Health University of Lincoln Brayford Pool Lincoln UK; ^3^ Department of Orthodontics Saveetha Dental College Chennai Tamil Nadu India

**Keywords:** knowledge synthesis, methodology, overview, systematic reviews

## Abstract

**Aim:**

The aim of this overview is to identify and collate evidence from existing published systematic review (SR) articles evaluating various methodological approaches used at each stage of an SR.

**Methods:**

The search was conducted in five electronic databases from inception to November 2020 and updated in February 2022: MEDLINE, Embase, Web of Science Core Collection, Cochrane Database of Systematic Reviews, and APA PsycINFO. Title and abstract screening were performed in two stages by one reviewer, supported by a second reviewer. Full‐text screening, data extraction, and quality appraisal were performed by two reviewers independently. The quality of the included SRs was assessed using the AMSTAR 2 checklist.

**Results:**

The search retrieved 41,556 unique citations, of which 9 SRs were deemed eligible for inclusion in final synthesis. Included SRs evaluated 24 unique methodological approaches used for defining the review scope and eligibility, literature search, screening, data extraction, and quality appraisal in the SR process. Limited evidence supports the following (a) searching multiple resources (electronic databases, handsearching, and reference lists) to identify relevant literature; (b) excluding non‐English, gray, and unpublished literature, and (c) use of text‐mining approaches during title and abstract screening.

**Conclusion:**

The overview identified limited SR‐level evidence on various methodological approaches currently employed during five of the seven fundamental steps in the SR process, as well as some methodological modifications currently used in expedited SRs. Overall, findings of this overview highlight the dearth of published SRs focused on SR methodologies and this warrants future work in this area.

## 1 INTRODUCTION

Evidence synthesis is a prerequisite for knowledge translation.^1^ A well conducted systematic review (SR), often in conjunction with meta‐analyses (MA) when appropriate, is considered the “gold standard” of methods for synthesizing evidence related to a topic of interest.^2^ The central strength of an SR is the transparency of the methods used to systematically search, appraise, and synthesize the available evidence.^3^ Several guidelines, developed by various organizations, are available for the conduct of an SR;^4^, ^5^, ^6^, ^7^ among these, Cochrane is considered a pioneer in developing rigorous and highly structured methodology for the conduct of SRs.^8^ The guidelines developed by these organizations outline seven fundamental steps required in SR process: defining the scope of the review and eligibility criteria, literature searching and retrieval, selecting eligible studies, extracting relevant data, assessing risk of bias (RoB) in included studies, synthesizing results, and assessing certainty of evidence (CoE) and presenting findings.^4^, ^5^, ^6^, ^7^

The methodological rigor involved in an SR can require a significant amount of time and resource, which may not always be available.^9^ As a result, there has been a proliferation of modifications made to the traditional SR process, such as refining, shortening, bypassing, or omitting one or more steps,^10^, ^11^ for example, limits on the number and type of databases searched, limits on publication date, language, and types of studies included, and limiting to one reviewer for screening and selection of studies, as opposed to two or more reviewers.^10^, ^11^ These methodological modifications are made to accommodate the needs of and resource constraints of the reviewers and stakeholders (e.g., organizations, policymakers, health care professionals, and other knowledge users). While such modifications are considered time and resource efficient, they may introduce bias in the review process reducing their usefulness.^5^

Substantial research has been conducted examining various approaches used in the standardized SR methodology and their impact on the validity of SR results. There are a number of published reviews examining the approaches or modifications corresponding to single^12^, ^13^ or multiple steps^14^ involved in an SR. However, there is yet to be a comprehensive summary of the SR‐level evidence for all the seven fundamental steps in an SR. Such a holistic evidence synthesis will provide an empirical basis to confirm the validity of current accepted practices in the conduct of SRs. Furthermore, sometimes there is a balance that needs to be achieved between the resource availability and the need to synthesize the evidence in the best way possible, given the constraints. This evidence base will also inform the choice of modifications to be made to the SR methods, as well as the potential impact of these modifications on the SR results. An overview is considered the choice of approach for summarizing existing evidence on a broad topic, directing the reader to evidence, or highlighting the gaps in evidence, where the evidence is derived exclusively from SRs.^15^ Therefore, for this review, an overview approach was used to (a) identify and collate evidence from existing published SR articles evaluating various methodological approaches employed in each of the seven fundamental steps of an SR and (b) highlight both the gaps in the current research and the potential areas for future research on the methods employed in SRs.

## 2 METHODS

An a priori protocol was developed for this overview but was not registered with the International Prospective Register of Systematic Reviews (PROSPERO), as the review was primarily methodological in nature and did not meet PROSPERO eligibility criteria for registration. The protocol is available from the corresponding author upon reasonable request. This overview was conducted based on the guidelines for the conduct of overviews as outlined in The Cochrane Handbook.^15^ Reporting followed the Preferred Reporting Items for Systematic reviews and Meta‐analyses (PRISMA) statement.^3^

### 2.1 Eligibility criteria

Only published SRs, with or without associated MA, were included in this overview. We adopted the defining characteristics of SRs from The Cochrane Handbook.^5^ According to The Cochrane Handbook, a review was considered systematic if it satisfied the following criteria: (a) clearly states the objectives and eligibility criteria for study inclusion; (b) provides reproducible methodology; (c) includes a systematic search to identify all eligible studies; (d) reports assessment of validity of findings of included studies (e.g., RoB assessment of the included studies); (e) systematically presents all the characteristics or findings of the included studies.^5^ Reviews that did not meet all of the above criteria were not considered a SR for this study and were excluded. MA‐only articles were included if it was mentioned that the MA was based on an SR.

SRs and/or MA of primary studies evaluating methodological approaches used in defining review scope and study eligibility, literature search, study selection, data extraction, RoB assessment, data synthesis, and CoE assessment and reporting were included. The methodological approaches examined in these SRs and/or MA can also be related to the substeps or elements of these steps; for example, applying limits on date or type of publication are the elements of literature search. Included SRs examined or compared various aspects of a method or methods, and the associated factors, including but not limited to: precision or effectiveness; accuracy or reliability; impact on the SR and/or MA results; reproducibility of an SR steps or bias occurred; time and/or resource efficiency. SRs assessing the methodological quality of SRs (e.g., adherence to reporting guidelines), evaluating techniques for building search strategies or the use of specific database filters (e.g., use of Boolean operators or search filters for randomized controlled trials), examining various tools used for RoB or CoE assessment (e.g., ROBINS vs. Cochrane RoB tool), or evaluating statistical techniques used in meta‐analyses were excluded.^14^

### 2.2 Search

The search for published SRs was performed on the following scientific databases initially from inception to third week of November 2020 and updated in the last week of February 2022: MEDLINE (via Ovid), Embase (via Ovid), Web of Science Core Collection, Cochrane Database of Systematic Reviews, and American Psychological Association (APA) PsycINFO. Search was restricted to English language publications. Following the objectives of this study, study design filters within databases were used to restrict the search to SRs and MA, where available. The reference lists of included SRs were also searched for potentially relevant publications.

The search terms included keywords, truncations, and subject headings for the key concepts in the review question: SRs and/or MA, methods, and evaluation. Some of the terms were adopted from the search strategy used in a previous review by Robson et al., which reviewed primary studies on methodological approaches used in study selection, data extraction, and quality appraisal steps of SR process.^14^ Individual search strategies were developed for respective databases by combining the search terms using appropriate proximity and Boolean operators, along with the related subject headings in order to identify SRs and/or MA.^16^, ^17^ A senior librarian was consulted in the design of the search terms and strategy. Appendix A presents the detailed search strategies for all five databases.

### 2.3 Study selection and data extraction

Title and abstract screening of references were performed in three steps. First, one reviewer (PV) screened all the titles and excluded obviously irrelevant citations, for example, articles on topics not related to SRs, non‐SR publications (such as randomized controlled trials, observational studies, scoping reviews, etc.). Next, from the remaining citations, a random sample of 200 titles and abstracts were screened against the predefined eligibility criteria by two reviewers (PV and MM), independently, in duplicate. Discrepancies were discussed and resolved by consensus. This step ensured that the responses of the two reviewers were calibrated for consistency in the application of the eligibility criteria in the screening process. Finally, all the remaining titles and abstracts were reviewed by a single “calibrated” reviewer (PV) to identify potential full‐text records. Full‐text screening was performed by at least two authors independently (PV screened all the records, and duplicate assessment was conducted by MM, HC, or MG), with discrepancies resolved via discussions or by consulting a third reviewer.

Data related to review characteristics, results, key findings, and conclusions were extracted by at least two reviewers independently (PV performed data extraction for all the reviews and duplicate extraction was performed by AP, HC, or MG).

### 2.4 Quality assessment of included reviews

The quality assessment of the included SRs was performed using the AMSTAR 2 (A MeaSurement Tool to Assess systematic Reviews). The tool consists of a 16‐item checklist addressing critical and noncritical domains.^18^ For the purpose of this study, the domain related to MA was reclassified from critical to noncritical, as SRs with and without MA were included. The other six critical domains were used according to the tool guidelines.^18^ Two reviewers (PV and AP) independently responded to each of the 16 items in the checklist with either “yes,” “partial yes,” or “no.” Based on the interpretations of the critical and noncritical domains, the overall quality of the review was rated as high, moderate, low, or critically low.^18^ Disagreements were resolved through discussion or by consulting a third reviewer.

### 2.5 Data synthesis

To provide an understandable summary of existing evidence syntheses, characteristics of the methods evaluated in the included SRs were examined and key findings were categorized and presented based on the corresponding step in the SR process. The categories of key elements within each step were discussed and agreed by the authors. Results of the included reviews were tabulated and summarized descriptively, along with a discussion on any overlap in the primary studies.^15^ No quantitative analyses of the data were performed.

## 3 RESULTS

**FIGURE 1:**
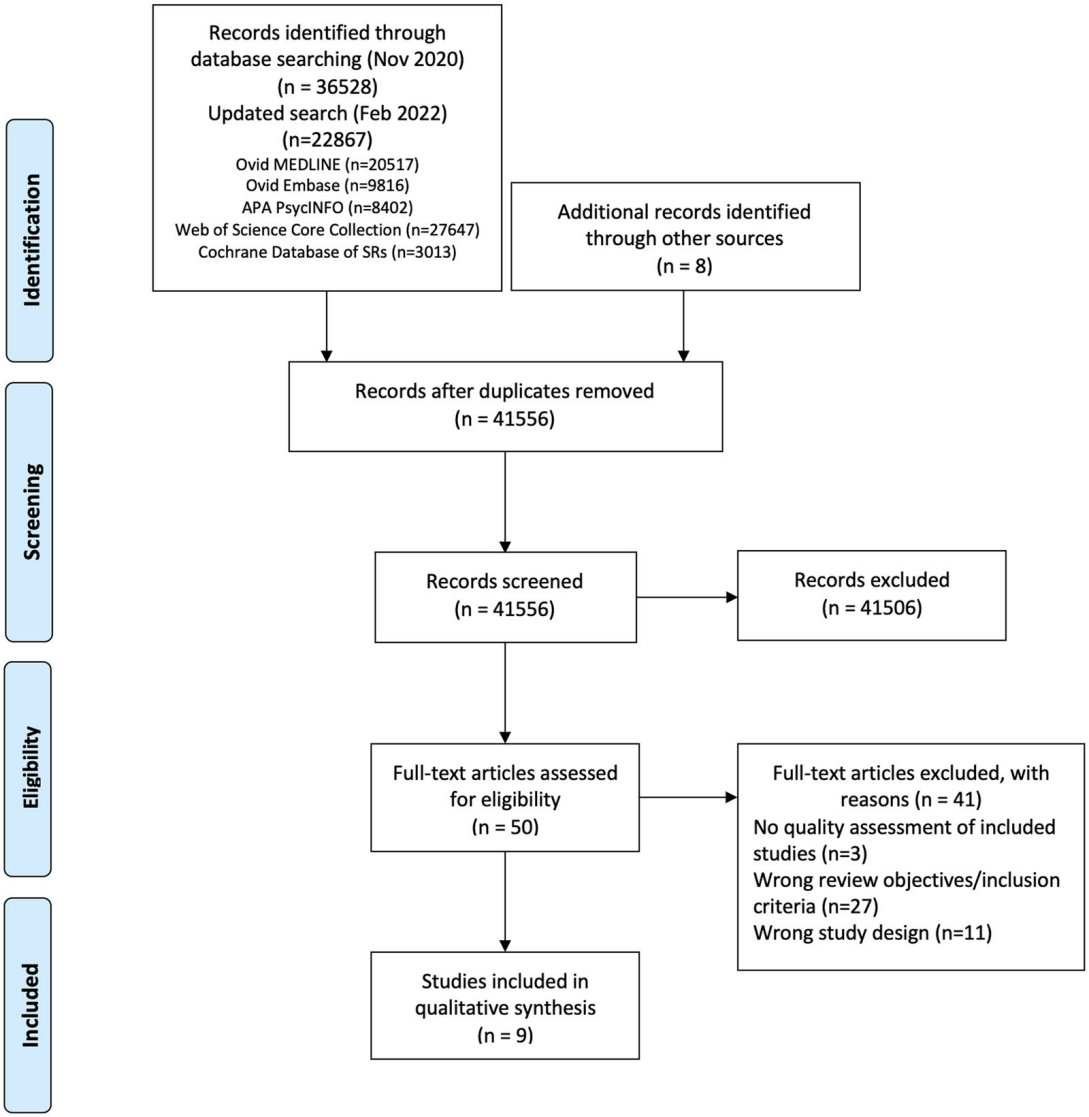
Study selection flowchart

From 41,556 unique citations identified through literature search, 50 full‐text records were reviewed, and nine systematic reviews^14^, ^19^, ^20^, ^21^, ^22^, ^23^, ^24^, ^25^, ^26^ were deemed eligible for inclusion. The flow of studies through the screening process is presented in Figure 1. A list of excluded studies with reasons can be found in Appendix B.

### 3.1 Characteristics of included reviews

Table 1 summarizes the characteristics of included SRs. The majority of the included reviews (six of nine) were published after 2010.^14^, ^22^, ^23^, ^24^, ^25^, ^26^ Four of the nine included SRs were Cochrane reviews.^20^, ^21^, ^22^, ^23^ The number of databases searched in the reviews ranged from 2 to 14, 2 reviews searched gray literature sources,^24^, ^25^ and 7 reviews included a supplementary search strategy to identify relevant literature.^14^, ^19^, ^20^, ^21^, ^22^, ^23^, ^26^ Three of the included SRs (all Cochrane reviews) included an integrated MA.^20^, ^21^, ^23^

**TABLE 1:** Characteristics of included studies

Author, year	Search strategy (year last searched; no. databases; supplementary searches)	SR design (type of review; no. of studies included)	Topic; subject area	SR objectives	SR authors’ comments on study quality
Crumley, 2005^19^	2004; Seven databases; four journals handsearched, reference lists and contacting authors	SR; *n* = 64	RCTs and CCTs; not specified	To identify and quantitatively review studies comparing two or more different resources (e.g., databases, Internet, handsearching) used to identify RCTs and CCTs for systematic reviews.	Most of the studies adequately described reproducible search methods, expected search yield. Poor quality in studies was mainly due to lack of rigor in reporting selection methodology. Majority of the studies did not indicate the number of people involved in independently screening the searches or applying eligibility criteria to identify potentially relevant studies.
Hopewell, 2007^20^	2002; eight databases; selected journals and published abstracts handsearched, and contacting authors	SR and MA; *n* = 34 (34 in quantitative analysis)	RCTs; health care	To review systematically empirical studies, which have compared the results of handsearching with the results of searching one or more electronic databases to identify reports of randomized trials.	The electronic search was designed and carried out appropriately in majority of the studies, while the appropriateness of handsearching was unclear in half the studies because of limited information. The screening studies methods used in both groups were comparable in most of the studies.
Hopewell, 2007 ^21^	2005; two databases; selected journals and published abstracts handsearched, reference lists, citations and contacting authors	SR and MA; *n* = 5 (5 in quantitative analysis)	RCTs; health care	To review systematically research studies, which have investigated the impact of gray literature in meta‐analyses of randomized trials of health care interventions.	In majority of the studies, electronic searches were designed and conducted appropriately, and the selection of studies for eligibility was similar for handsearching and database searching. Insufficient data for most studies to assess the appropriateness of handsearching and investigator agreeability on the eligibility of the trial reports.
Horsley, 2011^22^	2008; three databases; reference lists, citations and contacting authors	SR; *n* = 12	Any topic or study area	To investigate the effectiveness of checking reference lists for the identification of additional, relevant studies for systematic reviews. Effectiveness is defined as the proportion of relevant studies identified by review authors solely by checking reference lists.	Interpretability and generalizability of included studies was difficult. Extensive heterogeneity among the studies in the number and type of databases used. Lack of control in majority of the studies related to the quality and comprehensiveness of searching.
Morrison, 2012^24^	2011; six databases and gray literature	SR; *n* = 5	RCTs; conventional medicine	To examine the impact of English language restriction on systematic review‐based meta‐analyses	The included studies were assessed to have good reporting quality and validity of results. Methodological issues were mainly noted in the areas of sample power calculation and distribution of confounders.
Robson, 2019^14^	2016; three databases; reference lists and contacting authors	SR; *n* = 37	N/R	To identify and summarize studies assessing methodologies for study selection, data abstraction, or quality appraisal in systematic reviews.	The quality of the included studies was generally low. Only one study was assessed as having low RoB across all four domains. Majority of the studies were assessed to having unclear RoB across one or more domains.
Schmucker, 2017^26^	2016; four databases; reference lists	SR; *n* = 10	Study data; medicine	To assess whether the inclusion of data that were not published at all and/or published only in the gray literature influences pooled effect estimates in meta‐analyses and leads to different interpretation.	Majority of the included studies could not be judged on the adequacy of matching or adjusting for confounders of the gray/unpublished data in comparison to published data.
					Also, generalizability of results was low or unclear in four research projects
Morissette, 2011^23^	2009; five databases; reference lists and contacting authors	SR and MA; *n* = 6 (5 included in quantitative analysis)	N/R	To determine whether blinded versus unblinded assessments of risk of bias result in similar or systematically different assessments in studies included in a systematic review.	Four studies had unclear risk of bias, while two studies had high risk of bias.
O'Mara‐Eves, 2015^25^	2013; 14 databases and gray literature	SR; *n* = 44	N/R	To gather and present the available research evidence on existing methods for text mining related to the title and abstract screening stage in a systematic review, including the performance metrics used to evaluate these technologies.	Quality appraised based on two criteria‐sampling of test cases and adequacy of methods description for replication. No study was excluded based on the quality (author contact).

SR = systematic review; MA = meta‐analysis; RCT = randomized controlled trial; CCT = controlled clinical trial; N/R = not reported.

The included SRs evaluated 24 unique methodological approaches (26 in total) used across five steps in the SR process; 8 SRs evaluated 6 approaches,^19^, ^20^, ^21^, ^22^, ^23^, ^24^, ^25^, ^26^ while 1 review evaluated 18 approaches.^14^ Exclusion of gray or unpublished literature^21^, ^26^ and blinding of reviewers for RoB assessment^14^, ^23^ were evaluated in two reviews each. Included SRs evaluated methods used in five different steps in the SR process, including methods used in defining the scope of review (*n* = 3), literature search (*n* = 3), study selection (*n* = 2), data extraction (*n* = 1), and RoB assessment (*n* = 2) (Table 2).

**TABLE 2:** Summary of findings from review evaluating systematic review methods

Key elements	Author, year	Method assessed	Evaluations/outcomes (P—primary; S—secondary)	Summary of SR authors’ conclusions	Quality of review
**Step: Defining the review scope and eligibility**					
Excluding study data based on publication status	Hopewell, 2007^21^	Gray vs. published literature	Pooled effect estimate	Published trials are usually larger and show an overall greater treatment effect than gray trials. Excluding trials reported in gray literature from SRs and MAs may exaggerate the results.	Moderate
	Schmucker, 2017^26^	Gray and/or unpublished vs. published literature	P: Pooled effect estimate	Excluding unpublished trials had no or only a small effect on the pooled estimates of treatment effects. Insufficient evidence to conclude the impact of including unpublished or gray study data on MA conclusions.	Moderate
			S: Impact on interpretation of MA		
Excluding study data based on language of publication	Morrison, 2012^24^	English language vs. non‐English language publications	P: Bias in summary treatment effects	No evidence of a systematic bias from the use of English language restrictions in systematic review‐based meta‐analyses in conventional medicine. Conflicting results on the methodological and reporting quality of English and non‐English language RCTs. Further research required.	Low
			S: number of included studies and patients, methodological quality and statistical heterogeneity		
**Step: Searching for studies**					
Resources searching	Crumley, 2005^19^	Two or more resources^a^ searching vs. resource‐specific searching	Recall and precision	Multiple‐source comprehensive searches are necessary to identify all RCTs for a systematic review. For electronic databases, using the Cochrane HSS or complex search strategy in consultation with a librarian is recommended.	Critically low
Supplementary searching	Hopewell, 2007^20^	Handsearching only vs. one or more electronic database(s)^b^ searching	Number of identified randomized trials	Handsearching is important for identifying trial reports for inclusion in systematic reviews of health care interventions published in nonindexed journals. Where time and resources are limited, majority of the full English‐language trial reports can be identified using a complex search or the Cochrane HSS.	Moderate
	Horsley, 2011^22^	Checking reference list (no comparison)	P: additional yield of checking reference lists	There is some evidence to support the use of checking reference lists to complement literature search in systematic reviews.	Low
			S: additional yield by publication type, study design or both and data pertaining to costs		
**Step: Selecting studies**					
Reviewer characteristics	Robson, 2019^14^	Single vs. double reviewer screening	P: Accuracy, reliability, or efficiency of a method	Using two reviewers for screening is recommended. If resources are limited, one reviewer can screen, and other reviewer can verify the list of excluded studies.	Low
			S: factors affecting accuracy or reliability of a method		
		Experienced vs. inexperienced reviewers for screening		Screening must be performed by experienced reviewers	
		Screening by blinded vs. unblinded reviewers		Authors do not recommend blinding of reviewers during screening as the blinding process was time‐consuming and had little impact on the results of MA	
Use of technology for study selection	Robson, 2019^14^	Use of dual computer monitors vs. nonuse of dual monitors for screening	P: Accuracy, reliability, or efficiency of a method	There are no significant differences in the time spent on abstract or full‐text screening with the use and nonuse of dual monitors	Low
			S: factors affecting accuracy or reliability of a method		
		Use of Google translate to translate non‐English citations to facilitate screening		Use of Google translate to screen German language citations	
	O'Mara‐Eves, 2015^25^	Use of text mining for title and abstract screening	Any evaluation concerning workload reduction	Text mining approaches can be used to reduce the number of studies to be screened, increase the rate of screening, improve the workflow with screening prioritization, and replace the second reviewer. The evaluated approaches reported saving a workload of between 30% and 70%	Critically low
Order of screening	Robson, 2019^14^	Title‐first screening vs. title‐and‐abstract simultaneous screening	P: Accuracy, reliability, or efficiency of a method	Title‐first screening showed no substantial gain in time when compared to simultaneous title and abstract screening.	Low
			S: factors affecting accuracy or reliability of a method		
**Step: Data extraction**					
Reviewer characteristics	Robson, 2019^14^	Single vs. double reviewer data extraction	P: Accuracy, reliability, or efficiency of a method	Use two reviewers for data extraction. Single reviewer data extraction followed by the verification of outcome data by a second reviewer (where statistical analysis is planned), if resources preclude	Low
			S: factors affecting accuracy or reliability of a method		
		Experienced vs. inexperienced reviewers for data extraction		Experienced reviewers must be used for extracting continuous outcomes data	
		Data extraction by blinded vs. unblinded reviewers		Authors do not recommend blinding of reviewers during data extraction as it had no impact on the results of MA	
Use of technology for data extraction		Use of dual computer monitors vs. nonuse of dual monitors for data extraction		Using two computer monitors may improve the efficiency of data extraction	
		Data extraction by two English reviewers using Google translate vs. data extraction by two reviewers fluent in respective languages		Google translate provides limited accuracy for data extraction	
		Computer‐assisted vs. double reviewer extraction of graphical data		Use of computer‐assisted programs to extract graphical data	
Obtaining additional data		Contacting study authors for additional data		Recommend contacting authors for obtaining additional relevant data	
**Step: RoB assessment**					
Reviewer characteristics	Robson, 2019^14^	Quality appraisal by blinded vs. unblinded reviewers	P: Accuracy, reliability, or efficiency of a method	Inconsistent results on RoB assessments performed by blinded and unblinded reviewers. Blinding reviewers for quality appraisal not recommended	Low
			S: factors affecting accuracy or reliability of a method		
	Morissette, 2011^23^	Risk of bias (RoB) assessment by blinded vs. unblinded reviewers	P: Mean difference and 95% confidence interval between RoB assessment scores	Findings related to the difference between blinded and unblinded RoB assessments are inconsistent from the studies. Pooled effects show no differences in RoB assessments for assessments completed in a blinded or unblinded manner.	Moderate
			S: qualitative level of agreement, mean RoB scores and measures of variance for the results of the RoB assessments, and inter‐rater reliability between blinded and unblinded reviewers		
	Robson, 2019^14^	Experienced vs. inexperienced reviewers for quality appraisal	P: Accuracy, reliability, or efficiency of a method	Reviewers performing quality appraisal must be trained. Quality assessment tool must be pilot tested.	Low
			S: factors affecting accuracy or reliability of a method		
		Use of additional guidance vs. nonuse of additional guidance for quality appraisal		Providing guidance and decision rules for quality appraisal improved the inter‐rater reliability in RoB assessments.	
Obtaining additional data		Contacting study authors for obtaining additional information/use of supplementary information available in the published trials vs. no additional information for quality appraisal		Additional data related to study quality obtained by contacting study authors improved the quality assessment.	
RoB assessment of qualitative studies		Structured vs. unstructured appraisal of qualitative research studies		Use of structured tool if qualitative and quantitative studies designs are included in the review. For qualitative reviews, either structured or unstructured quality appraisal tool can be used.	

^a^ Includes databases (MEDLINE, Embase, PyscINFO, CINAHL, Biosis, CancerLIT, Cabnar, CENTRAL, Chirolars, HealthStar, SciCitIndex, Cochrane Central Trial Register), internet, and handsearching.

^b^ Includes MEDLINE, Embase, PsychLIT, PsychINFO, Lilac and Cochrane Central Trials Register; HSS‐Highly Sensitive Search; SR, systematic review; MA, meta‐analysis; RCT, randomized controlled trial; RoB, risk of bias.

There was some overlap in the primary studies evaluated in the included SRs on the same topics: Schmucker et al.^26^ and Hopewell et al.^21^ (*n* = 4), Hopewell et al.^20^ and Crumley et al.^19^ (*n* = 30), and Robson et al.^14^ and Morissette et al.^23^ (*n* = 4). There were no conflicting results between any of the identified SRs on the same topic.

### 3.2 Methodological quality of included reviews

Overall, the quality of the included reviews was assessed as moderate at best (Table 2). The most common critical weakness in the reviews was failure to provide justification for excluding individual studies (four reviews). Detailed quality assessment is provided in Appendix C.

### 3.3 Evidence on systematic review methods

#### 3.3.1 Methods for defining review scope and eligibility

Two SRs investigated the effect of excluding data obtained from gray or unpublished sources on the pooled effect estimates of MA.^21^, ^26^ Hopewell et al.^21^ reviewed five studies that compared the impact of gray literature on the results of a cohort of MA of RCTs in health care interventions. Gray literature was defined as information published in “print or electronic sources not controlled by commercial or academic publishers.” Findings showed an overall greater treatment effect for published trials than trials reported in gray literature. In a more recent review, Schmucker et al.^26^ addressed similar objectives, by investigating gray and unpublished data in medicine. In addition to gray literature, defined similar to the previous review by Hopewell et al., the authors also evaluated unpublished data—defined as “supplemental unpublished data related to published trials, data obtained from the Food and Drug Administration  or other regulatory websites or postmarketing analyses hidden from the public.” The review found that in majority of the MA, excluding gray literature had little or no effect on the pooled effect estimates. The evidence was limited to conclude if the data from gray and unpublished literature had an impact on the conclusions of MA.^26^

Morrison et al.^24^ examined five studies measuring the effect of excluding non‐English language RCTs on the summary treatment effects of SR‐based MA in various fields of conventional medicine. Although none of the included studies reported major difference in the treatment effect estimates between English only and non‐English inclusive MA, the review found inconsistent evidence regarding the methodological and reporting quality of English and non‐English trials.^24^ As such, there might be a risk of introducing “language bias” when excluding non‐English language RCTs. The authors also noted that the numbers of non‐English trials vary across medical specialties, as does the impact of these trials on MA results. Based on these findings, Morrison et al.^24^ conclude that literature searches must include non‐English studies when resources and time are available to minimize the risk of introducing “language bias.”

#### 3.3.2 Methods for searching studies

Crumley et al.^19^ analyzed recall (also referred to as “sensitivity” by some researchers; defined as “percentage of relevant studies identified by the search”) and precision (defined as “percentage of studies identified by the search that were relevant”) when searching a single resource to identify randomized controlled trials and controlled clinical trials, as opposed to searching multiple resources. The studies included in their review frequently compared a MEDLINE only search with the search involving a combination of other resources. The review found low median recall estimates (median values between 24% and 92%) and very low median precisions (median values between 0% and 49%) for most of the electronic databases when searched singularly.^19^ A between‐database comparison, based on the type of search strategy used, showed better recall and precision for complex and Cochrane Highly Sensitive search strategies (CHSSS). In conclusion, the authors emphasize that literature searches for trials in SRs must include multiple sources.^19^

In an SR comparing handsearching and electronic database searching, Hopewell et al.^20^ found that handsearching retrieved more relevant RCTs (retrieval rate of 92%−100%) than searching in a single electronic database (retrieval rates of 67% for PsycINFO/PsycLIT, 55% for MEDLINE, and 49% for Embase). The retrieval rates varied depending on the quality of handsearching, type of electronic search strategy used (e.g., simple, complex or CHSSS), and type of trial reports searched (e.g., full reports, conference abstracts, etc.). The authors concluded that handsearching was particularly important in identifying full trials published in nonindexed journals and in languages other than English, as well as those published as abstracts and letters.^20^

The effectiveness of checking reference lists to retrieve additional relevant studies for an SR was investigated by Horsley et al.^22^ The review reported that checking reference lists yielded 2.5%–40% more studies depending on the quality and comprehensiveness of the electronic search used. The authors conclude that there is some evidence, although from poor quality studies, to support use of checking reference lists to supplement database searching.^22^

#### 3.3.3 Methods for selecting studies

Three approaches relevant to reviewer characteristics, including number, experience, and blinding of reviewers involved in the screening process were highlighted in an SR by Robson et al.^14^ Based on the retrieved evidence, the authors recommended that two independent, experienced, and unblinded reviewers be involved in study selection.^14^ A modified approach has also been suggested by the review authors, where one reviewer screens and the other reviewer verifies the list of excluded studies, when the resources are limited. It should be noted however this suggestion is likely based on the authors’ opinion, as there was no evidence related to this from the studies included in the review.

Robson et al.^14^ also reported two methods describing the use of technology for screening studies: use of Google Translate for translating languages (for example, German language articles to English) to facilitate screening was considered a viable method, while using two computer monitors for screening did not increase the screening efficiency in SR. Title‐first screening was found to be more efficient than simultaneous screening of titles and abstracts, although the gain in time with the former method was lesser than the latter. Therefore, considering that the search results are routinely exported as titles and abstracts, Robson et al.^14^ recommend screening titles and abstracts simultaneously. However, the authors note that these conclusions were based on very limited number (in most instances one study per method) of low‐quality studies.^14^

#### 3.3.4 Methods for data extraction

Robson et al.^14^ examined three approaches for data extraction relevant to reviewer characteristics, including number, experience, and blinding of reviewers (similar to the study selection step). Although based on limited evidence from a small number of studies, the authors recommended use of two experienced and unblinded reviewers for data extraction. The experience of the reviewers was suggested to be especially important when extracting continuous outcomes (or quantitative) data. However, when the resources are limited, data extraction by one reviewer and a verification of the outcomes data by a second reviewer was recommended.

As for the methods involving use of technology, Robson et al.^14^ identified limited evidence on the use of two monitors to improve the data extraction efficiency and computer‐assisted programs for graphical data extraction. However, use of Google Translate for data extraction in non‐English articles was not considered to be viable.^14^ In the same review, Robson et al.^14^ identified evidence supporting contacting authors for obtaining additional relevant data.

#### 3.3.5 Methods for RoB assessment

Two SRs examined the impact of blinding of reviewers for RoB assessments.^14^, ^23^ Morissette et al.^23^ investigated the mean differences between the blinded and unblinded RoB assessment scores and found inconsistent differences among the included studies providing no definitive conclusions. Similar conclusions were drawn in a more recent review by Robson et al.,^14^ which included four studies on reviewer blinding for RoB assessment that completely overlapped with Morissette et al.^23^

Use of experienced reviewers and provision of additional guidance for RoB assessment were examined by Robson et al.^14^ The review concluded that providing intensive training and guidance on assessing studies reporting insufficient data to the reviewers improves RoB assessments.^14^ Obtaining additional data related to quality assessment by contacting study authors was also found to help the RoB assessments, although based on limited evidence. When assessing the qualitative or mixed method reviews, Robson et al.^14^ recommends the use of a structured RoB tool as opposed to an unstructured tool. No SRs were identified on data synthesis and CoE assessment and reporting steps.

## 4 DISCUSSION

### 4.1 Summary of findings

Nine SRs examining 24 unique methods used across five steps in the SR process were identified in this overview. The collective evidence supports some current traditional and modified SR practices, while challenging other approaches. However, the quality of the included reviews was assessed to be moderate at best and in the majority of the included SRs, evidence related to the evaluated methods was obtained from very limited numbers of primary studies. As such, the interpretations from these SRs should be made cautiously.

The evidence gathered from the included SRs corroborate a few current SR approaches.^5^ For example, it is important to search multiple resources for identifying relevant trials (RCTs and/or CCTs). The resources must include a combination of electronic database searching, handsearching, and reference lists of retrieved articles.^5^ However, no SRs have been identified that evaluated the impact of the number of electronic databases searched. A recent study by Halladay et al.^27^ found that articles on therapeutic intervention, retrieved by searching databases other than PubMed (including Embase), contributed only a small amount of information to the MA and also had a minimal impact on the MA results. The authors concluded that when the resources are limited and when large number of studies are expected to be retrieved for the SR or MA, PubMed‐only search can yield reliable results.^27^

Findings from the included SRs also reiterate some methodological modifications currently employed to “expedite” the SR process.^10^, ^11^ For example, excluding non‐English language trials and gray/unpublished trials from MA have been shown to have minimal or no impact on the results of MA.^24^, ^26^ However, the efficiency of these SR methods, in terms of time and the resources used, have not been evaluated in the included SRs.^24^, ^26^ Of the SRs included, only two have focused on the aspect of efficiency^14^, ^25^; O'Mara‐Eves et al.^25^ report some evidence to support the use of text‐mining approaches for title and abstract screening in order to increase the rate of screening. Moreover, only one included SR^14^ considered primary studies that evaluated reliability (inter‐ or intra‐reviewer consistency) and accuracy (validity when compared against a “gold standard” method) of the SR methods. This can be attributed to the limited number of primary studies that evaluated these outcomes when evaluating the SR methods.^14^ Lack of outcome measures related to reliability, accuracy, and efficiency precludes making definitive recommendations on the use of these methods/modifications. Future research studies must focus on these outcomes.

Some evaluated methods may be relevant to multiple steps; for example, exclusions based on publication status (gray/unpublished literature) and language of publication (non‐English language studies) can be outlined in the a priori eligibility criteria or can be incorporated as search limits in the search strategy. SRs included in this overview focused on the effect of study exclusions on pooled treatment effect estimates or MA conclusions. Excluding studies from the search results, after conducting a comprehensive search, based on different eligibility criteria may yield different results when compared to the results obtained when limiting the search itself.^28^ Further studies are required to examine this aspect.

Although we acknowledge the lack of standardized quality assessment tools for methodological study designs, we adhered to the Cochrane criteria for identifying SRs in this overview. This was done to ensure consistency in the quality of the included evidence. As a result, we excluded three reviews that did not provide any form of discussion on the quality of the included studies. The methods investigated in these reviews concern supplementary search,^29^ data extraction,^12^ and screening.^13^ However, methods reported in two of these three reviews, by Mathes et al.^12^ and Waffenschmidt et al.,^13^ have also been examined in the SR by Robson et al.,^14^ which was included in this overview; in most instances (with the exception of one study included in Mathes et al.^12^ and Waffenschmidt et al.^13^ each), the studies examined in these excluded reviews overlapped with those in the SR by Robson et al.^14^

One of the key gaps in the knowledge observed in this overview was the dearth of SRs on the methods used in the data synthesis component of SR. Narrative and quantitative syntheses are the two most commonly used approaches for synthesizing data in evidence synthesis.^5^ There are some published studies on the proposed indications and implications of these two approaches.^30^, ^31^ These studies found that both data synthesis methods produced comparable results and have their own advantages, suggesting that the choice of the method must be based on the purpose of the review.^31^ With increasing number of “expedited” SR approaches (so called “rapid reviews”) avoiding MA,^10^, ^11^ further research studies are warranted in this area to determine the impact of the type of data synthesis on the results of the SR.

### 4.2 Implications for future research

The findings of this overview highlight several areas of paucity in primary research and evidence synthesis on SR methods. First, no SRs were identified on methods used in two important components of the SR process, including data synthesis and CoE and reporting. As for the included SRs, a limited number of evaluation studies have been identified for several methods. This indicates that further research is required to corroborate many of the methods recommended in current SR guidelines.^4^, ^5^, ^6^, ^7^ Second, some SRs evaluated the impact of methods on the results of quantitative synthesis and MA conclusions. Future research studies must also focus on the interpretations of SR results.^28^, ^32^ Finally, most of the included SRs were conducted on specific topics related to the field of health care, limiting the generalizability of the findings to other areas. It is important that future research studies evaluating evidence syntheses broaden the objectives and include studies on different topics within the field of health care.

### 4.3 Strengths and limitations

To our knowledge, this is the first overview summarizing current evidence from SRs and MA on different methodological approaches used in several fundamental steps in SR conduct. The overview methodology followed well established guidelines and strict criteria defined for the inclusion of SRs.

There are several limitations related to the nature of the included reviews. Evidence for most of the methods investigated in the included reviews was derived from a limited number of primary studies. Also, the majority of the included SRs may be considered outdated as they were published (or last updated) more than 5 years ago^33^; only three of the nine SRs have been published in the last 5 years.^14^, ^25^, ^26^ Therefore, important and recent evidence related to these topics may not have been included. Substantial numbers of included SRs were conducted in the field of health, which may limit the generalizability of the findings. Some method evaluations in the included SRs focused on quantitative analyses components and MA conclusions only. As such, the applicability of these findings to SR more broadly is still unclear.^28^ Considering the methodological nature of our overview, limiting the inclusion of SRs according to the Cochrane criteria might have resulted in missing some relevant evidence from those reviews without a quality assessment component.^12^, ^13^, ^29^ Although the included SRs performed some form of quality appraisal of the included studies, most of them did not use a standardized RoB tool, which may impact the confidence in their conclusions. Due to the type of outcome measures used for the method evaluations in the primary studies and the included SRs, some of the identified methods have not been validated against a reference standard.

Some limitations in the overview process must be noted. While our literature search was exhaustive covering five bibliographic databases and supplementary search of reference lists, no gray sources or other evidence resources were searched. Also, the search was primarily conducted in health databases, which might have resulted in missing SRs published in other fields. Moreover, only English language SRs were included for feasibility. As the literature search retrieved large number of citations (i.e., 41,556), the title and abstract screening was performed by a single reviewer, calibrated for consistency in the screening process by another reviewer, owing to time and resource limitations. These might have potentially resulted in some errors when retrieving and selecting relevant SRs. The SR methods were grouped based on key elements of each recommended SR step, as agreed by the authors. This categorization pertains to the identified set of methods and should be considered subjective.

## 5 CONCLUSIONS

This overview identified limited SR‐level evidence on various methodological approaches currently employed during five of the seven fundamental steps in the SR process. Limited evidence was also identified on some methodological modifications currently used to expedite the SR process. Overall, findings highlight the dearth of SRs on SR methodologies, warranting further work to confirm several current recommendations on conventional and expedited SR processes.

## CONFLICT OF INTEREST

The authors declare no conflicts of interest.

## Supporting information

APPENDIX A: Detailed search strategies
APPENDIX B: List of excluded studies with detailed reasons for exclusion
APPENDIX C: Quality assessment of included reviews using AMSTAR 2

## References

[1] Ioannidis JPA . Evolution and translation of research findings: from bench to where. PLoS Clin Trials. 2006;1(7), e36. 17111044 10.1371/journal.pctr.0010036 PMC1851723

[2] Crocetti E . Systematic reviews with meta‐analysis:why, when, and how? Emerg Adulthood. 2016;4(1):3–18.

[3] Moher D , Liberati A , Tetzlaff J , Altman DG , The PG . Preferred reporting items for systematic reviews and meta‐analyses: the PRISMA statement. PLoS Med. 2009;6(7), e1000097. 19621072 10.1371/journal.pmed.1000097 PMC2707599

[4] Akers J . Systematic Reviews: CRD's Guidance for Undertaking Reviews in Health Care. CRD, University of York; 2009.

[5] Higgins JPT , Thomas J , Chandler J , et al., eds. Cochrane Handbook for Systematic Reviews of Interventions Version 6.3. Cochrane; 2022. http://www.training.cochrane.org/handbook. [updated February 2022]. Available from.

[6] Joanna Briggs Institute . Joanna Briggs Institute Reviewers’ Manual: 2015 Edition/Supplement. The Joanna Briggs Institute; 2015.

[7] Methods Group of the Campbell Collaboration . *Methodological expectations of Campbell Collaboration intervention reviews: Conduct standards*. 2016.

[8] Chandler J , Hopewell S . Cochrane methods—twenty years experience in developing systematic review methods. Syst Rev. 2013;2(1):76. 24050381 10.1186/2046-4053-2-76 PMC3849105

[9] Tsertsvadze A , Chen Y‐F , Moher D , Sutcliffe P , McCarthy N . How to conduct systematic reviews more expeditiously? Syst Rev. 2015;4: 160–160. 26563648 10.1186/s13643-015-0147-7 PMC4643500

[10] Ganann R , Ciliska D , Thomas H . Expediting systematic reviews: methods and implications of rapid reviews. Implement Sci. 2010;5: 56. 20642853 10.1186/1748-5908-5-56 PMC2914085

[11] Tricco AC , Antony J , Zarin W , et al. A scoping review of rapid review methods. BMC Med. 2015;13(1):224. 26377409 10.1186/s12916-015-0465-6 PMC4574114

[12] Mathes T , Klasen P , Pieper D . Frequency of data extraction errors and methods to increase data extraction quality: a methodological review. BMC Med Res Methodol. 2017;17(1):152. 29179685 10.1186/s12874-017-0431-4 PMC5704562

[13] Waffenschmidt S , Knelangen M , Sieben W , Buhn S , Pieper D . Single screening versus conventional double screening for study selection in systematic reviews: a methodological systematic review. BMC Med Res Methodol. 2019;19:132. 31253092 10.1186/s12874-019-0782-0 PMC6599339

[14] Robson RC , Pham B , Hwee J , et al. Few studies exist examining methods for selecting studies, abstracting data, and appraising quality in a systematic review. J Clin Epidemiol. 2019;106: 121–135. 30312656 10.1016/j.jclinepi.2018.10.003

[15] Pollock M , Fernandes RM , Becker LA , Pieper D , Hartling L . Chapter V: overviews of reviews. In: Higgins JPT , Thomas J , Chandler J , et al., eds. Cochrane Handbook for Systematic Reviews of Interventions version 6.3 (updated February 2022). Cochrane; 2022. Available from: http://www.training.cochrane.org/handbook

[16] Montori VM , Wilczynski NL , Morgan D , Haynes RB . Optimal search strategies for retrieving systematic reviews from Medline: analytical survey. BMJ. 2005;330(7482):68. 15619601 10.1136/bmj.38336.804167.47 PMC543864

[17] Wilczynski NL , Haynes RB . EMBASE search strategies achieved high sensitivity and specificity for retrieving methodologically sound systematic reviews. J Clin Epidemiol. 2007;60(1):29–33. 17161751 10.1016/j.jclinepi.2006.04.001

[18] Shea BJ , Reeves BC , Wells G , et al. AMSTAR 2: a critical appraisal tool for systematic reviews that include randomised or non‐randomised studies of healthcare interventions, or both. BMJ. 2017;358: j4008. 28935701 10.1136/bmj.j4008 PMC5833365

[19] Crumley ET , Wiebe N , Cramer K , Klassen TP , Hartling L . Which resources should be used to identify RCT/CCTs for systematic reviews: a systematic review. BMC Med Res Methodol. 2005;5: 24. 16092960 10.1186/1471-2288-5-24 PMC1232852

[20] Hopewell S , Clarke MJ , Lefebvre C , Scherer RW . Handsearching versus electronic searching to identify reports of randomized trials. Cochrane Database Syst Rev. 2007;2007(2), MR000001. 10.1002/14651858.MR000001.pub2 PMC7437388 17443625

[21] Hopewell S , McDonald S , Clarke M , Egger M . Grey literature in meta‐analyses of randomized trials of health care interventions. Cochrane Database Syst Rev. 2007(2), MR000010. 17443631 10.1002/14651858.MR000010.pub3 PMC8973936

[22] Horsley T , Dingwall O , Sampson M . Checking reference lists to find additional studies for systematic reviews. Cochrane Database Syst Rev. 2011;2011(8), MR000026. 10.1002/14651858.MR000026.pub2 PMC7388740 21833989

[23] Morissette K , Tricco AC , Horsley T , Chen MH , Moher D . Blinded versus unblinded assessments of risk of bias in studies included in a systematic review. Cochrane Database Syst Rev. 2011;2011(9), MR000025. 10.1002/14651858.MR000025.pub2 PMC7433288 21901737

[24] Morrison A , Polisena J , Husereau D , et al. The effect of English‐language restriction on systematic review‐based meta‐analyses: a systematic review of empirical studies. Int J Technol Assess Health Care. 2012;28(2):138–144. 22559755 10.1017/S0266462312000086

[25] O'Mara‐Eves A , Thomas J , McNaught J , Miwa M , Ananiadou S . Using text mining for study identification in systematic reviews: a systematic review of current approaches. Syst Rev. 2015;4(1):5. 25588314 10.1186/2046-4053-4-5 PMC4320539

[26] Schmucker CM , Blumle A , Schell LK , et al. Systematic review finds that study data not published in full text articles have unclear impact on meta‐analyses results in medical research. PLoS ONE [Electronic Resource]. 2017;12(4), e0176210. 10.1371/journal.pone.0176210 PMC5404772 28441452

[27] Halladay CW , Trikalinos TA , Schmid IT , Schmid CH , Dahabreh IJ . Using data sources beyond PubMed has a modest impact on the results of systematic reviews of therapeutic interventions. J Clin Epidemiol. 2015;68(9):1076–1084. 26279401 10.1016/j.jclinepi.2014.12.017

[28] Nussbaumer‐Streit B , Klerings I , Dobrescu A , et al. Excluding non‐English publications from evidence‐syntheses did not change conclusions: a meta‐epidemiological study. J Clin Epidemiol. 2020;118: 42–54. 31698064 10.1016/j.jclinepi.2019.10.011

[29] Cooper C , Booth A , Britten N , Garside R . A comparison of results of empirical studies of supplementary search techniques and recommendations in review methodology handbooks: a methodological review. Syst Rev. 2017;6(1):234. 29179733 10.1186/s13643-017-0625-1 PMC5704629

[30] Melendez‐Torres GJ , O'Mara‐Eves A , Thomas J , Brunton G , Caird J , Petticrew M . Interpretive analysis of 85 systematic reviews suggests that narrative syntheses and meta‐analyses are incommensurate in argumentation. Res Synth Methods. 2017;8(1):109–118. 27860329 10.1002/jrsm.1231 PMC5347877

[31] Melendez‐Torres GJ , Thomas J , Lorenc T , O'Mara‐Eves A , Petticrew M . Just how plain are plain tobacco packs: re‐analysis of a systematic review using multilevel meta‐analysis suggests lessons about the comparative benefits of synthesis methods. Syst Rev. 2018;7(1):153. 30290842 10.1186/s13643-018-0821-7 PMC6173910

[32] Nussbaumer‐Streit B , Klerings I , Wagner G , et al. Abbreviated literature searches were viable alternatives to comprehensive searches: a meta‐epidemiological study. J Clin Epidemiol. 2018;102: 1–11. 29864540 10.1016/j.jclinepi.2018.05.022

[33] Shojania KG , Sampson M , Ansari MT , Ji J , Doucette S , Moher D . How quickly do systematic reviews go out of date? A survival analysis. Ann Intern Med. 2007;147(4):224–233. 17638714 10.7326/0003-4819-147-4-200708210-00179

