# Supplementary material for: An overview of methodological approaches in systematic reviews
Source: J Evid Based Med. 2022 Apr 13;15(1):39–54. doi: 10.1111/jebm.12468 (PMC9322259; doi:10.1111/jebm.12468)
Supplement: Supplementary file 1 — APPENDIX A: Detailed search strategies APPENDIX B: List of excluded studies with detailed reasons for exclusion APPENDIX C: Quality assessment of included reviews using AMSTAR 2 [file JEBM-15-39-s001.docx]

**Appendix A: Detailed search strategies**

**Database: MEDLINE (Ovid)**

**Search date: <1946 to 20 November 2020> Updated on 28 February 2022**

1 Meta-Analysis as Topic/ 18597

2 exp Review literature as Topic/ 14814

3 (meta-analy* or metaanaly* or meta analy* or systematic review?).tw,pt. 317026

4 limit 3 to yr="2019-current" 79867

5 1 or 2 or 4 108582

6 "Information Storage and Retrieval"/ 20248

7 Databases, Bibliographic/ 6020

8 Gray Literature/ 21

9 ((bibliographic* or citation* or data or database* or grey or gray or information

or literature) adj2 (retriev* or search* or source* or stor*)).tw. 206463

10 ((bibliographic* or citation* or reference or references) adj2 manag*).tw. 523

11 ((review* or screen* or select*) adj2 (abstract* or bibliograph* or citation* or

data or record* or study or studies)).tw. 209835

12 ((reviewer* or screener*) adj2 number*).tw. 73

13 ((study or record* or article or design or paper or trial) adj (selection or identification or inclusion)).tw. 20895

14 ((inclusion or exclusion or eligib* or screen*) adj2 (criteri* or study or studies)).tw. 173360

15 ((data or stud* or record* or reviewer* or abstract or title or reliability or

eligibility or accuracy) adj2 screen*).tw. 29282

16 Data Collection/ 89862

17 "Abstracting and Indexing as Topic"/ 4771

18 (data adj2 (abstract* or analy* or extract* or handl*)).tw. 356681

19 exp *Bias/ 6631

20 ((assess* or apprais* or estimat* or grade* or grading) adj2 (bias* or risk)).tw. 143934

21 ((assess* or apprais* or grade* or grading) adj2 (critical* or methodolog*

or quality or stud*)).tw. 219121

22 (("quality appraisal" or "critical appraisal" or "quality assessment") adj

(tool* or checklist* or scale*)).tw. 2819

23 (reporting adj3 guideline*).tw. 3149

24 (report* adj1 (findings or result*)).tw. 34555

25 (CONSORT or ENTREQ or MOOSE or PRISMA or QUOROM or STROBE).tw. 19045

26 ((data or evidence or narrative) adj synthes*).tw. 19581

27 or/6-26 1234288

28 (impact adj2 (results or result or conclusion or conclusions)).tw. 4528

29 ((inconsistenc* or discordan* or discrepan*) adj result*).tw. 5112

30 Efficiency/ 13898

31 (efficien* or productiv*).tw. 1082481

32 Workload/ 21693

33 (workload* or work load*).tw. 32546

34 Time Factors/ 1195130

35 (time sav* or time requir* or resources consum* or resource* or sav* or cost*).tw. 650270

36 Reproducibility of Results/ 404337

37 ((reproducib* or reliabilit* or reliabl* or replicab* or replicat* or valid*) adj

(results or findings or outcomes)).tw. 16085

38 Data Accuracy/ 2698

39 "Sensitivity and Specificity"/ 349789

40 (data adj2 (accurat* or accuracy or exact* or precis*)).tw. 11617

41 (method* adj2 (accurat* or accuracy or correct* or evaluat* or guid* or

quality or standard* or impact or efficien*)).tw. 224252

42 (methodological adj3 (consider* or decision* or design* or issue* or quality or alter* or modif*)).tw. 29733

43 ((inter-rater* or intra-rater* or interrater* or intrarater* or inter-reviewer* or intra-reviewer* or interreviewer* or intrareviewer* or observer* or inter-observer* or interobserver* or intra-observer* or intraobserver*) adj3 (agree* or varia* or reliability or validity)).tw. 44343

44 or/28-43 3577854

45 5 and 27 and 44 12563

46 limit 45 to dt=20201111-20220228 7954

**Database: Embase (Ovid)**

**Search date: <1947 to 20 November 2020> Updated on 28 February 2022**

1 exp "meta analysis (topic)"/ 43765

2 exp "systematic review (topic)"/ 25647

3 (meta-analy* or metaanaly* or meta analy* or systematic review*).kw,pt. 81438

4 limit 3 to yr="2019-current" 21910

5 1 or 2 or 4 80515

6 information retrieval/ 39556

7 data base/ 239566

8 ((bibliographic* or citation* or data or database* or grey or gray or information or

literature) adj2 (retriev* or search* or source* or stor*)).kw,ti,ab. 263317

9 ((bibliographic* or citation* or reference or references) adj2 manag*).kw,ti,ab. 756

10 ((review* or screen* or select*) adj2 (abstract* or bibliograph* or citation* or

data or record* or study or studies)).kw,ti,ab. 299239

11 ((reviewer* or screener*) adj2 number*).kw,ti,ab. 92

12 ((study or record* or article or design or paper or trial) adj (selection or

identification or inclusion)).kw,ti,ab. 29802

13 ((inclusion or exclusion or eligib* or screen*) adj2 (criteri* or study or studies)).kw,ti,ab. 296119

14 ((data or stud* or record* or reviewer* or abstract or title or reliability or

eligibility or accuracy) adj2 screen*).kw,ti,ab. 43683

15 information processing/ 245759

16 (data adj2 (abstract* or analy* or extract* or handl*)).kw,ti,ab. 534040

17 "risk of bias".kw,ti,ab. 28493

18 ((assess* or apprais* or estimat* or grade* or grading) adj2 (bias* or risk)).kw,ti,ab. 207808

19 ((assess* or apprais* or grade* or grading) adj2 (critical* or methodolog* or

quality or stud*)).kw,ti,ab. 302719

20 (("quality appraisal" or "critical appraisal" or "quality assessment") adj

(tool* or checklist* or scale*)).kw,ti,ab. 3492

21 (reporting adj3 guideline*).kw,ti,ab. 4117

22 (report* adj1 (findings or result*)).kw,ti,ab. 67229

23 (CONSORT or ENTREQ or MOOSE or PRISMA or QUOROM or STROBE).kw,ti,ab. 24761

24 ((data or evidence or narrative) adj synthes*).kw,ti,ab. 23351

25 or/6-24 2073570

26 (impact adj2 (results or result or conclusion or conclusions)).kw,ti,ab. 8378

27 ((inconsistenc* or discordan* or discrepan*) adj result*).kw,ti,ab. 8789

28 productivity/ 43005

29 (efficien* or productiv*).kw,ti,ab. 1326410

30 workload/ 46575

31 (workload* or work load*).kw,ti,ab. 47367

32 time factor/ 37323

33 (time sav* or time requir* or resources consum* or resource* sav* or cost*).kw,ti,ab. 903850

34 reproducibility/ 225012

35 ((reproducib* or replicab* or replicat* or valid*) adj (results or findings or outcomes)).kw,ti,ab. 15169

36 data accuracy/ 539

37 "sensitivity and specificity"/ 376433

38 (data adj2 (accurat* or accurac* or exact* or precis*)).kw,ti,ab. 16310

39 (method* adj2 (accurat* or accurac* or correct* or evaluat* or guid* or

quality or standard* or impact or efficien*)).kw,ti,ab. 330338

40 (methodological adj3 (consider* or decision* or design* or issue* or

quality or alter* or modif*)).kw,ti,ab. 35567

41 ((inter-rater* or intra-rater* or interrater* or intrarater* or inter-reviewer* or

intra-reviewer* or interreviewer* or intrareviewer* or observer* or inter-observer* or

interobserver* or intra-observer* or intraobserver*) adj3 (agree* or varia* or reliability or validity)).kw,ti,ab. 61625

42 or/26-41 3047283

43 5 and 25 and 42 6629

44 limit 43 to dc=20201120-20220228 3187

**Database: APA PsycInfo**

**Search date: <1806 to 20 November 2020> Updated on 28 February 2022**

1 meta analysis/ 4896

2 "literature review"/ 22802

3 (meta-analy* or metaanaly* or meta analy* or systematic review?).md,tw. 68490

4 1 or 2 or 3 90470

5 *databases/ 2662

6 ((bibliographic* or citation* or data or database* or grey or gray or

information or literature) adj2 (retriev* or search* or source* or stor*)).tw. 57442

7 ((bibliographic* or citation* or reference or references) adj2 manag*).tw. 142

8 ((review* or screen* or select*) adj2 (abstract* or bibliograph* or citation*

or data or record* or study or studies)).tw. 50070

9 ((reviewer* or screener*) adj2 number*).tw. 36

10 ((study or record* or article or design or paper or trial) adj

(selection or identification or inclusion)).tw. 2059

11 ((inclusion or exclusion or eligib* or screen*) adj2 (criteri* or study or studies)).tw. 23800

12 ((data or stud* or record* or reviewer* or abstract or title or reliability

or eligibility or accuracy) adj2 screen*).tw. 4393

13 *data collection/ 5058

14 (data adj2 (abstract* or analy* or extract* or handl*)).tw. 112352

15 ((assess* or apprais* or estimat* or grade* or grading) adj2 (bias* or risk)).tw. 24954

16 ((assess* or apprais* or grade* or grading) adj2 (critical* or methodolog* or

quality or stud*)).tw. 86060

17 (("quality appraisal" or "critical appraisal" or "quality assessment") adj

(tool* or checklist* or scale*)).tw. 509

18 (reporting adj3 guideline*).tw. 636

19 (report* adj1 (findings or result*)).tw. 13485

20 (CONSORT or ENTREQ or MOOSE or PRISMA or QUOROM or STROBE).tw. 3121

21 "synthes*".tw. 52167

22 or/5-21 380490

23 (impact adj2 (results or result or conclusion or conclusions)).tw. 1406

24 ((inconsistenc* or discordan* or discrepan*) adj result*).tw. 611

25 (efficien* or productiv*).tw. 119767

26 *work load/ 2275

27 (workload* or work load*).tw. 9296

28 (time sav* or time requir* or resources consum* or resource* sav* or cost*).tw. 113176

29 exp Test Reliability/ or exp Test Validity/ 92053

30 (reproducib* or reliabilit* or reliabl* or replicab* or replicat* or valid*).tw. 400523

31 (data adj2 (accurat* or accuracy or exact* or precis*)).tw. 1730

32 (method* adj2 (accurat* or accuracy or correct* or evaluat* or guid* or

quality or standard* or impact or efficien*)).tw. 28324

33 (methodological adj3 (consider* or decision* or design* or issue* or

quality or alter* or modif*)).tw. 19011

34 ((inter-rater* or intra-rater* or interrater* or intrarater* or inter-reviewer* or intra-reviewer* or interreviewer* or intrareviewer* or observer* or inter-observer* or interobserver* or intra-observer* or intraobserver*) adj3 (agree* or varia* or reliability or validity)).tw. 10714

35 or/23-34 647651

36 4 and 22 and 35 10058

37 limit 36 to ("0830 systematic review" or 1200 meta analysis) 7269

38 limit 37 to up=20201120-20220228 1133

**Database: Web of Science (core collection)**

**Search date: <1900 to 16 November 2020> Updated on 28 February 2022**

32 #31 and LD=(2020-11-16/2022-02-28) 10312

31 #1 and #28 and #29 and Review Articles (Document Types) 17335

30 #1 and #28 and #29 35732

29 #18 or #19 or #20 or #21 or #22 or #23 or #24 or #25 or #26 or #27 7554812

28 #2 or #3 or #4 or #5 or #6 or #7 or #8 or #9 or #10 or #11 or #12 or #13 or #14 or #15 or #16 or #17 3205453

27 TS=(((inter-rater* or intra-rater* or interrater* or intrarater* or inter-reviewer* or intra-reviewer* or interreviewer* or intrareviewer* or observer* or inter-observer* or interobserver* or

intra-observer* or intraobserver*) near/3 (agree* or varia* or reliability or validity))) 54962

26 TS=((methodological near/3 (consider* or decision* or design* or issue* or quality or alter* or modif*))) 44821

25 TS=((method* near/2 (accurat* or accurac* or correct* or evaluat* or guid* or quality

or standard* or impact or efficien*))) 745506

24 TS=((data near/2 (accurat* or accurac* or exact* or precis*))) 60455

23 TS=(((reproducib* or replicab* or replicat* or valid*) near (results or findings or outcomes))) 566098

22 TS=((time sav* or time requir* or resources consum* or resource* sav* or cost*)) 2935808

21 TS=((workload* or work load*)) 255305

20 TS=((efficien* or productiv*)) 3954566

19 TS=(((inconsistenc* or discordan* or discrepan*) near result*)) 45719

18 TS=(impact near/2 (results or result or conclusion or conclusions)) 37665

17 TS=(((data or evidence or narrative) near synthes*)) 92280

16 TS=((CONSORT or ENTREQ or MOOSE or PRISMA or QUOROM or STROBE)) 27212

15 TS=((report* near/1 (findings or result*))) 188649

14 TS=((reporting near/3 guideline*)) 8651

13 TS=((("quality appraisal" or "critical appraisal" or "quality assessment") near

(tool* or checklist* or scale*))) 7476

12 TS=(((assess* or apprais* or grade* or grading) near/2 (critical* or methodolog* or quality or stud*))) 578380

11 TS=(((assess* or apprais* or estimat* or grade* or grading) near/2 (bias* or risk))) 304108

10 TS=(risk of bias) 75877

9 TS=((data near/2 (abstract* or analy* or extract* or handl*))) 891258

8 TS=(((data or stud* or record* or reviewer* or abstract or title or reliability or

eligibility or accuracy) near/2 screen*)) 53849

7 TS=((inclusion or exclusion or eligib* or screen*) near/2 (criteri* or study or studies)) 204035

6 TS=(((study or record* or article or design or paper or trial) near

(selection or identification or inclusion))) 558730

5 TS=(((reviewer* or screener*) near/2 number*)) 185

4 TS=(((review* or screen* or select*) near/2 (abstract* or bibliograph* or citation* or

data or record* or study or studies))) 446669

3 TS=((bibliographic* or citation* or reference or references) near/2 manag*) 3389

2 TS=((bibliographic* or citation* or data or database* or grey or gray or information or

literature) near/2 (retriev* or search* or source* or stor*)) 490358

1 TS=(meta-analy* or metaanaly* or meta analy* or systematic review*) 583370

**Database: Cochrane Register for Systematic Reviews**

**Search date: 20 November 2020**

1 MeSH descriptor: [Meta-Analysis as Topic] this term only 285

2 MeSH descriptor: [Review Literature as Topic] explode all trees 126

3 ((meta-analy* or metaanaly* or meta analy* or systematic review?)):ti,ab,kw 25339

4 #1 or #2 or #3 25368

5 MeSH descriptor: [Information Storage and Retrieval] this term only 121

6 MeSH descriptor: [Databases, Bibliographic] explode all trees 68

7 ((bibliographic* or citation* or data or database* or grey or gray or information or literature)

NEAR/2 (retriev* or search* or source* or stor*)):ti,ab,kw 8656

8 ((bibliographic* or citation* or reference or references) NEAR/2 manag*):ti,ab,kw 29

9 ((review* or screen* or select*) NEAR/2 (abstract* or bibliograph* or citation* or

data or record* or study or studies)):ti,ab,kw 20122

10 ((reviewer* or screener*) NEAR/2 number*):ti,ab,kw 4

11 ((study or record* or article or design or paper or trial) NEAR

(selection or identification or inclusion)):ti,ab,kw 27456

12 ((inclusion or exclusion or eligib* or screen*) NEAR/2 (criteri* or study or studies)):ti,ab,kw. 192376

13 ((data or stud* or record* or reviewer* or abstract or title or

reliability or eligibility or accuracy) NEAR/2 screen*):ti,ab,kw 6290

14 MeSH descriptor: [Data Collection] this term only 1169

15 MeSH descriptor: [Abstracting and Indexing] this term only 23

16 ((data NEAR/2 (abstract* or analy* or extract* or handl*))):ti,ab,kw 60008

17 MeSH descriptor: [Bias] this term only 945

18 ((assess* or apprais* or estimat* or grade* or grading) NEAR/2 (bias* or risk)):ti,ab,kw 30962

19 ((assess* or apprais* or grade* or grading) NEAR/2

(critical* or methodolog* or quality or stud*)):ti,ab,kw 67676

20 (("quality appraisal" or "critical appraisal" or "quality assessment") NEAR

(tool* or checklist* or scale*)):ti,ab,kw 176

21 (reporting NEAR/3 guideline*):ti,ab,kw 245

22 (report* NEAR/1 (findings or result*)):ti,ab,kw 5354

23 (CONSORT or ENTREQ or MOOSE or PRISMA or QUOROM or STROBE):ti,ab,kw 1749

24 (synthes*):ti,ab,kw 11216

25 #5 or #6 or #7 or #8 or #9 or #10 or #11 or #12 or #13 or #14 or #15

or #16 or #17 or #18 or #19 or #20 or #21 or #22 or #23 or #24 356836

26 (impact NEAR/2 (results or result or conclusion or conclusions)):ti,ab,kw 1040

27 ((inconsistenc* or discordan* or discrepan*) NEAR result*):ti,ab,kw 969

28 MeSH descriptor: [Efficiency] this term only 344

29 (efficien* or productiv*):ti,ab,kw 34758

30 MeSH descriptor: [Workload] this term only 407

31 (workload* or work load*):ti,ab,kw 5595

32 MeSH descriptor: [Time Factors] this term only 66267

33 (time sav* or time requir* or resources consum* or resource* sav* or cost*):ti,ab,kw 126687

34 MeSH descriptor: [Reproducibility of Results] this term only 10999

35 ((reproducib* or reliabilit* or reliabl* or replicab* or replicat* or valid*)):ti,ab,kw 91200

36 MeSH descriptor: [Data Accuracy] this term only 30

37 MeSH descriptor: [Sensitivity and Specificity] this term only 8701

38 ((data NEAR/2 (accurat* or accuracy or exact* or precis*))):ti,ab,kw 603

39 (method* NEAR/2 (accurat* or accuracy or correct* or evaluat* or

guid* or quality or standard* or impact or efficien*)):ti,ab,kw 25160

40 (methodological NEAR/3 (consider* or decision* or design*

or issue* or quality or alter* or modif*)):ti,ab,kw 2923

41 ((inter-rater* or intra-rater* or interrater* or intrarater* or inter-reviewer* or

intra-reviewer* or interreviewer* or intrareviewer* or observer* or inter-observer* or interobserver*

or intra-observer* or intraobserver*) NEAR/3 (agree* or varia* or reliability or validity)):ti,ab,kw 5098

42 #26 or #27 or #28 or #29 or #30 or #31 or #32 or #33 or

#34 or #35 or #36 or #37 or #38 or #39 or #40 or #41 307893

43 #4 AND #25 AND #42 in Cochrane Reviews 2732

44 #43 with Cochrane Library publication date between 20 Nov 2020 and 28 Feb 2022 281

**Appendix B: List of excluded studies with detailed reasons for exclusion**

| **Study** | **Citation** | **Reasons for exclusion** |
| --- | --- | --- |
| Alpi 2019 | Alpi KM, Vo TA, Dorman DC. Language Consideration and Methodological Transparency in "Systematic" Reviews of Animal Toxicity Studies. International Journal of Toxicology 2019; 38(2): 135-45. | Wrong study design-primary research study |
| Arditi 2016 | Arditi C, Burnand B, Peytremann-Bridevaux I. Adding non-randomised studies to a Cochrane review brings complementary information for healthcare stakeholders: an augmented systematic review and meta-analysis. BMC Health Services Research 2016; 16. | Not a systematic review of primary studies |
| Bafeta 2013 | Bafeta A, Trinquart L, Seror R, Ravaud P. Analysis of the systematic reviews process in reports of network meta-analyses: methodological systematic review. Bmj-British Medical Journal 2013; 347. | Does not evaluate a systematic review method |
| Bagg 2020 | Bagg MK, O'Hagan E, Zahara P, et al. Systematic reviews that include only published data may overestimate the effectiveness of analgesic medicines for low back pain: a systematic review and meta-analysis. Journal of Clinical Epidemiology 2020; 124: 149-59. | Not a systematic review of primary studies |
| Bramer 2013 | Bramer WM, Giustini D, Kramer BMR, Anderson PF. The comparative recall of Google Scholar versus PubMed in identical searches for biomedical systematic reviews: a review of searches used in systematic reviews. Systematic Reviews 2013; 2. | Wrong study design-primary research study |
| Cooper 2017 | Cooper C, Booth A, Britten N, Garside R. A comparison of results of empirical studies of supplementary search techniques and recommendations in review methodology handbooks: a methodological review. Systematic Reviews 2017; 6(1): 234. | Not a systematic review- does not include a quality assessment of included studies |
| Doig 2003 | Doig GS, Simpson F. Efficient literature searching: a core skill for the practice of evidence-based medicine. Intensive Care Medicine 2003; 29(12): 2119-27. | Does not evaluate a systematic review method |
| Duyx 2017 | Duyx B, Urlings MJE, Swaen GMH, Bouter LM, Zeegers MP. Scientific citations favor positive results: a systematic review and meta-analysis. Journal of Clinical Epidemiology 2017; 88: 92-101. | Does not evaluate a systematic review method |
| Dwan 2011 | Dwan K, Altman DG, Cresswell L, Blundell M, Gamble CL, Williamson PR. Comparison of protocols and registry entries to published reports for randomised controlled trials. Cochrane Database of Systematic Reviews 2011; (1). | Does not evaluate a systematic review method |
| Dwan 2013 | Dwan K, Gamble C, Williamson PR, Kirkham JJ, Reporting Bias G. Systematic Review of the Empirical Evidence of Study Publication Bias and Outcome Reporting Bias - An Updated Review. PLoS ONE 2013; 8(7). | Does not evaluate a systematic review method |
| Feldmann 2019 | Feldmann J, Puhan MA, Mutsch M. Characteristics of stakeholder involvement in systematic and rapid reviews: A methodological review in the area of health services research. BMJ Open 2019; 9(8). | Does not evaluate a systematic review method |
| Furlan 2011 | Furlan JC, Singh J, Hsieh J, Fehlings MG. Methodology of systematic reviews and recommendations. Journal of Neurotrauma 2011; 28(8): 1335-9. | Not a systematic review of primary studies |
| Halladay 2015 | Halladay CW, Trikalinos TA, Schmid IT, Schmid CH, Dahabreh IJ. Using data sources beyond PubMed has a modest impact on the results of systematic reviews of therapeutic interventions. Journal of Clinical Epidemiology 2015; 68(9): 1076-84. | Wrong study design-primary research study |
| Hugues 2020 | Hugues A, Di Marco J, Bonan I, Rode G, Cucherat M, Gueyffier F. Publication language and the estimate of treatment effects of physical therapy on balance and postural control after stroke in meta-analyses of randomised controlled trials. PLoS ONE 2020; 15(3): e0229822. | Not a systematic review of primary studies |
| Johnson 2019 | Johnson BT, Hennessy EA. Systematic reviews and meta-analyses in the health sciences: Best practice methods for research syntheses. Social Science & Medicine 2019; 233: 237-51. | Not a systematic review of primary studies |
| Mathes 2017 | Mathes T, Klasen P, Pieper D. Frequency of data extraction errors and methods to increase data extraction quality: a methodological review. BMC Medical Research Methodology 2017; 17(1): 152. | Not a systematic review-does not include a quality assessment of included studies |
| Mayo-Wilson 2018 | Mayo-Wilson E, Li TJ, Fusco N, Dickersin K, Muds I. Practical guidance for using multiple data sources in systematic reviews and meta-analyses (with examples from the MUDS study). Research Synthesis Methods 2018; 9(1): 2-12. | Not a systematic review of primary studies |
| Mertz 2017 | Mertz M, Strech D, Kahrass H. What methods do reviews of normative ethics literature use for search, selection, analysis, and synthesis? In-depth results from a systematic review of reviews. Systematic Reviews 2017; 6(1): 261. | Not a systematic review of primary studies |
| Moher, 2000 | Moher D, Pham B, Klassen TP, et al. What contributions do languages other than English make on the results of meta-analyses? J Clin Epidemiol 2000; 53(9): 964-72. | Wrong study design-primary research study |
| Mueller, 2016 | Mueller KF, Meerpohl JJ, Briel M, et al. Methods for detecting, quantifying, and adjusting for dissemination bias in meta-analysis are described. Journal of Clinical Epidemiology 2016; 80: 25-33. | Does not evaluate a systematic review method |
| Orgeolet 2020 | Orgeolet L, Foulquier N, Misery L, et al. Can artificial intelligence replace manual search for systematic literature? Review on cutaneous manifestations in primary Sjogren's syndrome. Rheumatology 2020; 59(4): 811-9. | Not a systematic review of primary studies |
| Page 2014 | Page MJ, McKenzie JE, Kirkham J, et al. Bias due to selective inclusion and reporting of outcomes and analyses in systematic reviews of randomised trials of healthcare interventions. Cochrane Database of Systematic Reviews 2014; (10). | Does not evaluate a systematic review method |
| Page 2018 | Page MJ, McKenzie JE, Higgins JPT. Tools for assessing risk of reporting biases in studies and syntheses of studies: a systematic review. BMJ Open 2018; 8(3): e019703. | Does not evaluate a systematic review method |
| Paulsell 2017 | Paulsell D, Thomas J, Monahan S, Seftor NS. A Trusted Source of Information: How Systematic Reviews Can Support User Decisions About Adopting Evidence-Based Programs. Evaluation Review 2017; 41(1): 50-77. | Does not evaluate a systematic review method |
| Pham, 2005 | Pham B, Klassen TP, Lawson ML, Moher D. Language of publication restrictions in systematic reviews gave different results depending on whether the intervention was conventional or complementary. Journal of Clinical Epidemiology 2005; 58(8): 769-76.e2. | Wrong study design-primary research study |
| Polanin 2016 | Polanin JR, Tanner-Smith EE, Hennessy EA. Estimating the difference between published and unpublished effect sizes: A meta-review. Review of Educational Research 2016; 86(1): 207-36. | Not a systematic review of primary studies |
| Puljak 2020 | Puljak L, Riva N, Parmelli E, Gonzalez-Lorenzo M, Moja L, Pieper D. Data extraction methods: an analysis of internal reporting discrepancies in single manuscripts and practical advice. Journal of Clinical Epidemiology 2020; 117: 158-64. | Wrong study design-primary research study |
| Rice 2016 | Rice DB, Kloda LA, Levis B, Qi B, Kingsland E, Thombs BD. Are MEDLINE searches sufficient for systematic reviews and meta-analyses of the diagnostic accuracy of depression screening tools? A review of meta-analyses. J Psychosom Res 2016; 87: 7-13. | Not a systematic review of primary studies |
| Tricco 2008 | Tricco AC, Tetzlaff J, Sampson M, et al. Few systematic reviews exist documenting the extent of bias: a systematic review. Journal of Clinical Epidemiology 2008; 61(5): 422-34. | Does not evaluate a systematic review method |
| Tricco 2018 | Tricco AC, Zarin W, Ghassemi M, et al. Same family, different species: methodological conduct and quality varies according to purpose for five types of knowledge synthesis. Journal of Clinical Epidemiology 2018; 96: 133-42. | Does not evaluate a systematic review method |
| Tsafnat 2018 | Tsafnat G, Glasziou P, Karystianis G, Coiera E. Automated screening of research studies for systematic reviews using study characteristics. Systematic Reviews 2018; 7(1): 64. | Wrong study design-primary research study |
| Tudur Smith 2016 | Tudur Smith C, Marcucci M, Nolan SJ, Iorio A, Sudell M, Riley R, Rovers MM, Williamson PR. Individual participant data meta‐analyses compared with meta‐analyses based on aggregate data. Cochrane Database of Systematic Reviews 2016, Issue 9. | Does not evaluate a systematic review method |
| vanEnst 2014 | van Enst WA, Scholten RJ, Whiting P, Zwinderman AH, Hooft L. Meta-epidemiologic analysis indicates that MEDLINE searches are sufficient for diagnostic test accuracy systematic reviews. Journal of Clinical Epidemiology 2014; 67(11): 1192-9. | Not a systematic review of primary studies |
| Waffenschmidt 2019 | Waffenschmidt S, Knelangen M, Sieben W, Buhn S, Pieper D. Single screening versus conventional double screening for study selection in systematic reviews: a methodological systematic review. BMC Medical Research Methodology 2019; 19. | Not a systematic review-does not include a quality assessment of included studies |
| Weise 2020 | Weise A, Buchter R, Pieper D, Mathes T. Assessing Context Suitability (Generalisability, External Validity, Applicability or Transferability) of Findings in Evidence Syntheses in Healthcare - An Integrative Review of Methodological Guidance. Research synthesis methods 2020; 13. | Does not evaluate a systematic review method |
| Willis 2011 | Willis BH, Quigley M. Uptake of newer methodological developments and the deployment of meta-analysis in diagnostic test research: a systematic review. BMC Medical Research Methodology 2011; 11: 27. | Does not evaluate a systematic review method |
| Minozzi 2022 | Minozzi SK, Dwan F, Borrelli, Filippini G. Reliability of the revised Cochrane risk-of-bias tool for randomised trials (RoB2) improved with the use of implementation instruction. Journal of Clinical Epidemiology 2022; 141:99-105. | Wrong study design-primary research study |
| Gunnell 2022 | Gunnell KE, Belcourt VJ, Tomasone JR, Weeks LC. Systematic review methods. International Review of Sport and Exercise Psychology 2022; <https://doi.org/10.1080/1750984X.2021.1966823> | Wrong review objectives |
| de Kock 2020 | de Kock S, Stirk L, Ross J, Duffy S, Noake C, Misso K. Systematic review search methods evaluated using the Preferred Reporting of Items for Systematic Reviews and Meta-Analyses and the Risk of Bias in Systematic reviews tool. International Journal of Technology Assessment in Health Care 2020; 37:e18. | Wrong study design-primary research study |
| Bethel 2021 | Bethel AC, Rogers M, Abbott R. Use of a search summary table to improve systematic review search methods, results, and efficiency. Journal of the Medical Library Association 2021; 109 (1):97-106. | Wrong study design-primary research study |
| Gusenbauer 2020 | Gusenbauer M, Haddaway NR. Which academic search systems are suitable for systematic reviews or meta-analyses? Evaluating retrieval qualities of Google Scholar, PubMed, and 26 other resources. Research synthesis methods 2020; 11(2):181-217. | Wrong study design-primary research study |

**Appendix C: Quality assessment of included reviews using AMSTAR 2**

| **AMSTAR 2 domains** | **Crumley, 2005 (19)** | **Hopewell, 2007 (20)** | **Hopewell, 2007 (21)** | **Horsley, 2011 (22)** | **Morrison, 2012 (24)** | **O’Mara-Eves 2015 (25)** | **Robson, 2019 (14)** | **Schmucker, 2017 (26)** | **Morissette, 2011 (23)** |
| --- | --- | --- | --- | --- | --- | --- | --- | --- | --- |
| Did the research questions and inclusion criteria for the review include the components of PICO? | NA | NA | NA | NA | NA | NA | NA | NA | NA |
| Did the report of the review contain an  explicit statement that the review methods were established prior to the conduct of the review and did the report justify any significant deviations from the protocol? | N | Y | Y | Y | Y | P | Y | Y | Y |
| Did the review authors explain their selection of the study designs for inclusion in the review? | P | P | P | Y | Y | Y | P | Y | Y |
| Did the review authors use a comprehensive literature search strategy? | Y | Y | Y | Y | Y | Y | Y | Y | Y |
| Did the review authors perform study selection in duplicate? | Y | Y | Y | Y | Y | Y | Y | Y | Y |
| Did the review authors perform data extraction in duplicate? | N | Y | Y | N | Y | N | N | Y | Y |
| Did the review authors provide a list of excluded studies and justify the exclusions? | N | Y | Y | Y | N | N | Y | N | Y |
| Did the review authors describe the included studies in adequate detail? | Y | Y | Y | Y | Y | Y | Y | Y | Y |
| Did the review authors use a satisfactory technique for assessing the risk of bias (RoB) in individual studies that were included in the review? | Y | Y | Y | P | Y | Y | Y | Y | Y |
| Did the review authors report on the sources of funding for the studies included in the review? | Y | N | N | N | Y | N | N | N | Y |
| If meta‐analysis was performed did the  review authors use appropriate methods for statistical combination of results? | NA | Y | Y | NA | NA | NA | NA | NA | Y |
| If meta‐analysis was performed did the  review authors assess the potential impact of RoB in individual studies on the results of the meta‐analysis or other evidence synthesis? | NA | N | N | NA | NA | NA | NA | NA | P |
| Did the review authors account for RoB in individual studies when interpreting/discussing the results of the review? | Y | Y | Y | N | Y | N | Y | Y | Y |
| Did the review authors provide a satisfactory explanation for, and discussion of, any heterogeneity observed in the results of the review? | Y | N | N | N | Y | N | P | Y | Y |
| If they performed quantitative synthesis did the review authors carry out an adequate investigation of publication bias (small study bias) and discuss its likely impact on the results of the review? | NA | N | N | NA | NA | NA | NA | NA | N |
| Did the review authors report any potential sources of conflict of interest, including any funding they received for conducting the review? | Y | Y | Y | Y | Y | Y | Y | Y | Y |
| **Overall Quality Assessment** | Critically low | Moderate | Moderate | Low | Low | Critically low | Moderate | Low | Moderate |

Y=Yes, P= Partial yes, N= No, NA=Not applicable
